# Violence in primary care: Prevalence and follow-up of victims

**DOI:** 10.1186/1471-2296-7-15

**Published:** 2006-03-09

**Authors:** Claire Morier-Genoud, Patrick Bodenmann, Bernard Favrat, Marco Vannotti

**Affiliations:** 1 Rue de l'Ecole du Commerce 6 1004 Lausanne, Switzerland; 2 Medical Outpatient Clinic, University of Lausanne, Switzerland Rue du Bugnon, 44 1011 Lausanne, Switzerland

## Abstract

**Background:**

Primary care physicians underestimate the prevalence of domestic violence and community violence. Victims are therefore at risk of further episodes of violence, with psychological and physical consequences. We used an interview to assess the prevalence of domestic and community violence among Swiss natives and foreigners. In a follow-up study, we evaluated the consequences of the interview for the positive patients.

**Methods:**

We evaluated the prevalence of violence by use of a questionnaire in an interview, in an academic general internal medicine clinic in Switzerland. In a follow-up, we evaluated the consequences of the interview for positive patients. The participants were 38 residents and 446 consecutive patients. Questionnaires were presented in the principal language spoken by our patients. They addressed sociodemographics, present and past violence, the security or lack of security felt by victims of violence, and the patients' own violence. Between 3 and 6 months after the first interview, we did a follow-up of all patients who had reported domestic violence in the last year.

**Results:**

Of the 366 patients included in the study, 36 (9.8%) reported being victims of physical violence during the last year (physicians identified only 4 patients out of the 36), and 34/366 (9.3%) reported being victims of psychological violence. Domestic violence was responsible for 67.3% of the cases, and community violence for 21.8%. In 10.9% of the cases, both forms of violence were found.

Of 29 patients who reported being victims of domestic violence, 22 were found in the follow-up. The frequency of violence had diminished (4/22) or the violence had ceased (17/22).

**Conclusion:**

The prevalence of violence is high; domestic violence is more frequent than community violence. There was no statistically significant difference between the Swiss and foreign patients' responses related to the rates of violence. Patients in a currently violent relationship stated that participating in the study helped them and that the violence decreased or ceased a few months later.

## Background

Violence is defined by the World Health Organization (WHO) as "the intentional use of physical force or power, threatened or actual, against oneself, another person, or against a group or community, that either results in or has a high likelihood of resulting in injury, death, psychological harm, maldevelopment, or deprivation." Violence can be divided into three broad categories, which are self-directed violence, interpersonal violence, and collective violence. Our focus is on interpersonal violence, which is divided into two subcategories: family and intimate partner violence, and community violence; that is, violence between unrelated individuals who may or may not know each other. The nature of violent acts can be physical, sexual, or psychological, or involve deprivation or neglect [1]. UNICEF applies the term "domestic violence," which we will use, for family and intimate partner violence. It includes violence that is perpetrated by intimate partners and other family members, and that is manifested through physical abuse, sexual abuse, psychological abuse, economic abuse, and acts of omission [2].

According to the WHO, an estimated 1.6 million people from around the world died as a result of violence (homicide, suicide, and war-related violence), and 520,000 were killed as a result of interpersonal violence, in 2000. This is the "tip of the iceberg," as it is impossible to estimate non-fatal violence precisely [1]. Crime statistics from the Federal Police Office (OFP) of Switzerland show that bodily injuries have nearly doubled between 1982 and 2000, and that threats and constraints have increased fourfold. As for offenses against sexual integrity, their number has not clearly increased over the long term, and an important decrease was seen in 2000 [3]. The Law of Aid to Victims of Infractions (LAVI) consultation centers were established by the 1993 federal law on assistance for victims of offence to help them and their relatives. The centers registered 21,255 cases in 2001. Most consultations concerned violence against women (73%), and 52% of all assisted persons were aged under 30. Violence took place in the family setting in 48% of all cases and in an intimate relationship in 68% of them. Bodily injuries (34%) and sexual attacks (36%) were important types of violence encountered by these centers [4]. Moreover, in a survey of the health of the Swiss people, 10% of men and 8% of women reported that they had suffered at least one form of violence (verbal, physical or sexual, or crime against propriety) in the last 12 months [5].

As for domestic violence, which we know is frequent, especially towards women, 48 population-based surveys from around the world found that 10 to 69% of women reported being physically assaulted by an intimate male partner at some point in their lives [6]. A recent American survey showed that more than half of the surveyed women (51.9%) and men (66.4%) were physically assaulted as a child by an adult caretaker and/or as an adult by any type of attacker. This means that an estimated 1.9 million women and 3.2 million men are physically assaulted each year in the United States. As for intimate partner violence, 22.1% of women and 7.4% of men in this survey said they were physically assaulted by a current or former intimate partner at some time in their lives; the rates for the last 12 months were 1.3% for women and 0.9% for men [7]. In Switzerland, 6.1% of women were physically or sexually assaulted in the last 12 months; 12.6% of women suffered physical violence, and 11.6% suffered sexual violence at some time in their lives [8].

An estimate of the direct costs of violence concluded that 400 million Swiss francs are annually spent on violence against women by public authorities, through the health care system, police and judicial interventions, social services, shelters or specialized centers for battered women, and for research on the subject, most of it for intimate partner violence [9]. However, these estimates do not include indirect costs such as the loss of economic productivity from women [2].

Even though battered women seek more hospital care than other women [10,11], physicians report having very little exposure to battered women and they lack awareness of the prevalence of domestic violence against women. They also tend to exclude the care of battered women with no physical injury from their professional duties [12].

The aim of our study was to assess the prevalence of domestic and community violence among the patients (Swiss natives and foreigners) of an academic general internal medicine clinic. A follow-up was carried out to assess the consequences of the interview for patients who had reported domestic violence.

## Methods

Our study limits itself to psychological and physical abuse experienced during the last 12 months and during the patient's lifetime.

### • Design and setting

We conducted a study that allowed us to screen for violence among our patients, with a follow-up for patients who were screened as being victims or perpetrators of domestic violence. It took place in an academic general internal medicine clinic in Lausanne, Switzerland. The site is a primary care clinic where residents provide adult ambulatory care for approximately 22,000 outpatient visits per year, at walk-in and follow-up visits. We focused on walk-in patients, as they are not known beforehand by the residents. We included all physicians working at the clinic at the time of the study.

We based our sample-size calculation on a recent study using a similar design [13]. We based our calculation on an alpha of 5% and a power of 80%. We estimated the prevalence of violence among our patients at about 10%. This led to a required minimal sample size of 121 participants for the study. We did not consider a clustering effect in our sample-size calculation, because the correlation of patients within the physician's consultation would be too small, as shown previously in our institution [14].

### • Participants

The physicians were 22 women and 16 men. The majority of the residents were at the end of postgraduate training in general internal medicine or family medicine (median duration of training, 6 years).

We tried to minimize a Hawthorne effect; that is, a change in attitude in response to attention from the investigators (15). To blind them to the purpose of the study, we only informed them that a study focusing on the principal health problems brought up during consultations would take place among the patients. The physicians received a questionnaire through which they were asked about the main problems they identified during the consultation.

Consecutive patients (men and women) consulting for an emergency visit at the clinic were approached during business hours (8 h-18 h) on weekdays. Eligible patients were over 18 years of age and spoke French, or were able to read French, English, Spanish, or Albanian, the languages into which the questionnaire was translated, and agreed to be questioned alone. Patients who were severely ill and needed to lie down were not included in the study, as they could not be interviewed in a private place. A research psychologist informed the recruited patients that the study was aimed at improving the medical follow-up of patients. For safety reasons recommended by the World Health Organization [16], we told them of the actual subject of the study only when we met them in an isolated place, and we had a diversion questionnaire ready in case someone entered the room during the interview. Of the 446 consecutive patients approached, 366 agreed to participate. The sex distribution and mean age of patients who did not participate was not significantly different from those who participated. Patients screened as victims or perpetrators of violence were offered a brochure containing guidelines (advice, legal information, and useful addresses) on intimate-partner violence, and the psychologist made sure that the situation was taken care of, after the consultation. We blinded the physicians for the purpose of the study, apart from telling a physician after the consultation if a patient had a violence problem. The ethics committee of the University of Lausanne approved the study protocol.

### • Data collection

The psychologist interviewed patients before the medical visit. Patients were asked for demographic information, which included their age, sex, nationality, spoken language(s), level of education, profession, marital status, household composition, and family composition. The questionnaire itself comprised a modified version of the partner violence screen (PVS), a tool tested and validated in 1994 in two urban emergency departments in Denver, Colorado [17]. This questionnaire was piloted with 11 patients from the same clinical setting for organization, clarity and ease of completion. The questions were as follows: "Have you been hit, kicked, punched, or otherwise hurt by someone within the past year, including the last 24 hours? If so, by whom?"; "Do you feel safe with your partner or in your family circle?"; "Is there a partner or a member of your family circle, past or present, who is making you feel unsafe now?" Two questions addressing the problem of insecurity and the reason for the medical visit were included ("Has anybody threatened you or have you been forced to do anything during the last year, including the last 24 hours? If so, by whom?"; "Is your request for a consultation due to these circumstances?"). Also, as we were interested not only in victims but also in perpetrators of violence, we added three questions about a patient's own violence towards someone in their family circle ("Do you feel that, in a moment of nervous irritation or of illness, you have lost your temper towards someone in your family circle?"; "Have you at one time or another, in a moment of nervous irritation, hit or shouted at someone in your family circle?"; "Are you afraid of not being able to control yourself in a moment of nervous irritation?"). Finally, after the beginning of the study, because of frequent answers regarding violence at some other time in the patients' lives, we added one question to estimate the lifetime prevalence of violence.

Between 3 and 6 months after the intervention, we phoned the patients who had been screened as victims or perpetrators of violence to learn whether their situation had changed. The questionnaire assessed the frequency and form of violence suffered or perpetrated. We also asked them if they had talked about it with their partner or with someone else.

### • Statistical analysis

We used the chi-square or Fisher's exact test for categorical data and a *t*-test or Wilcoxon rank-sum test for continuous data. We considered p values < 0.05 to be significant.

To look for confounding and interaction in a multivariable analysis (regression analysis), we introduced age, sex, nationality, spoken language, level of education, profession, composition of the patient's household circle, and composition of the patient's family circle into the model.

## Results

### • Resident characteristics

The physicians were similar in their sociodemographic characteristics and professional achievements: they were 22 women and 16 men; their mean age was 32.9 years old. The majority of them were at the end of the postgraduate training in general internal medicine (median duration of training: 6 years). Among all residents, one of them came from Africa, another from Eastern- Europe, and all the others were Caucasian.

### • Patient characteristics

We approached 446 patients in total. The demographic characteristics of the patients were similar and are summarized in Table 1. Most patients used a French questionnaire, but 10.4% of them used a questionnaire in another language.

**Table 1:** Demographic characteristics

**Age **N = 446	Mean age 36.45 years; minimum age 16 (three 16-year-olds and two 17-year-olds); maximum age 91
**Sex **N = 446	217 men (48.7%) and 229 women (51.3%)
**Nationality **N = 446	67 nationalities: 153 (69%) Swiss patients (15 double nationals) and 293 (31%) foreign patients
**Nationality by region **N = 446	282 Europeans (10 double European nationals); 62 Africans; 33 Latin Americans; 32 from the Eastern Mediterranean; 11 South-East Asians; 10 double nationals (9 European + other and 1 non European); 9 from the Western Pacific; 7 North Americans
**Marital Status **N = 446	198 (45%) single; 178 (40%) married; 51 (11%) separated or divorced; 19 (4%) widowed
**Household composition **N = 367	124 (32%) persons were living alone; 104 (26%) with one other person; 62 (16%) with 2 persons; 48 (12%) with 3 persons; 21 (5%) with 4 persons; 8 (2%) with 5 to 7 persons; 15 (4%) in collocation with 2 or 3 persons and 10 (3%) were living in a shelter or mental hospital, or were homeless.
**Level of education **N = 388	0–4 years of school: 16 patients (4%); adapted training: 4 (1%); 5–8 years of schooling: 50 (13%); 9–14 years of schooling: 79 (20.5%); apprenticeship: 74 (19%); high school diploma: 46 (12%); professional school: 55 (14%); university or equivalent: 64 (16.5%)
**Professional activity **N = 445	Schooling/elementary training: 3 (1%); post-school training: 57 (13%); housewife: 36 (8%); unemployed: 94 (21%); retired: 28 (6%); unskilled worker: 121 (27%); skilled worker, junior executive: 72 (16%); middle or senior executive: 34 (8%)

### • Prevalence of violence

We found that 9.8% (36/366) of the patients had suffered physical violence (only 4 patients of these 36 were identified by physicians) and that 9.3% (34/366) had suffered psychological violence in the last 12 months, with 15 patients declaring both forms. Domestic violence was responsible for 67.3% (37 patients), and community violence for 21.8% (12 patients). In 10.9% (6 cases), both domestic violence and community violence were reported. Patients said they had suffered violence at another time in their lives in 49% (126/257) of the cases; the forms were domestic violence in 50.8% of the patients, community violence in 38.1%, and both forms in 11.1%.

The patients who were victims of physical or psychological violence were asked more questions. Some of them (15.8%, 15/95; some of the patients with past violence were included) said they did not feel safe with their partner or in their family circle, and 28.4% (27/95) said they currently felt unsafe, because of a past or present partner/member of their family circle. The request for a consultation was due to these circumstances in 10.3% (9/87) of the cases. Three questions about the perpetrator's position were asked. To the first question, 30.3% (111/366) of the patients answered in the affirmative. To the second question, 55.7% (204/366) of the patients said "yes" for shouting and 36.9% (135/366) for hitting. To the last one, 17.2% (63/366) answered "yes."

There was no significant difference between the responses of Swiss and foreign patients, except for one regarding the patients' own violence. More foreign patients than Swiss patients said they were afraid of not being able to control themselves (13 Swiss, 50 foreigners; p-value 0.0009). We also found a significant difference between men and women. Men suffer more community violence and women more domestic violence, as present and past violence experienced (present domestic violence: 5 men and 13 women; present community violence: 24 men and 13 women; p-value: 0.009; past domestic violence: 29 men and 49 women; past community violence: 30 men and 18 women; p-value: 0.05). These results are summarized in Table 2.

**Table 2:** Prevalence of violence

Patients who reported being **victims of physical violence in the last year**	9.8% (36/366)
Patients who reported being **victims of psychological violence in the last year**	9.3% (34/366)
Community violence	21.8%
Domestic violence	67.3%
Community and domestic violence	10.9%
**Lifetime violence **(at least once)	49% (126/257)
Community violence	38.1%
Domestic violence	50.8%
Community and domestic violence	11.1%
Not safe with partner or in family circle	15.8% (15/95)
Presently unsafe because of past/present partner/member of family circle	28.4% (27/95)
Request of consultation due to these circumstances	10.3% (9/87)

### • Follow-up of patients identified as victims/perpetrators of domestic violence

The patients who were screened as being victims or perpetrators of domestic violence were re-contacted by phone. They were 18 victims and 16 perpetrators of violence; 5 were both. The victims were 5 men and 13 women; their mean age was 29.44 (range, 18–44 years of age); 10 were from Europe (8 being Swiss), 5 were from Latin America (one being a double national: European and Latin American), and 4 were from Africa. The perpetrators were 8 men and 8 women; their mean age was 33.12 (range, 21–59 years of age); there were 7 from Europe (3 being Swiss), 1 from North America, 5 from Latin America, and 4 from Africa. We were able to make contact with 22 of the 29 patients involved; 3 victims and 4 perpetrators could not be found. More than half the victims (60%; 9/15) and perpetrators (58.3%; 7/12) said they had been able to talk about their problem of violence with the person responsible for the violence or with the victim; 73.3% (11/15) of the victims and 41.7% (5/12) of the perpetrators spoke with someone else. The violence totally ceased for 73.3% (11/15) of the victims and 75% (9/12) of the perpetrators. It diminished in frequency for 20% (3/15) of the victims and 16.7% (2/12) of the perpetrators, and in the case of one victim/perpetrator, it occurred once between her participation in the study and the follow-up interview. The form of violence changed for 46.7% (7/15) of the victims and 45.5% (5/11) of the perpetrators, mostly becoming verbal (6/15 of the victims; 5/11 of the perpetrators). One victim lost the possibility of seeing his children anymore. Interestingly enough, some patients (10/22; 45.5%; victims and perpetrators together) told us, without us asking, that the study helped them by allowing them to talk about the violence or in becoming conscious that the situation should be changed, etc. Finally, it seems important to state that for 7 of the patients (31.8%), the relationship they had at the time of the study had ended by the time we did this follow-up. The results are summarized in Table 3.

**Table 3:** Follow-up of patients having been identified as victims/perpetrators of domestic violence

Patients found at follow-up	22/29	
	**Victims**	**Perpetrators**

Able to talk about problem of violence with perpetrator or victim	60% (9/15)	58.3% (7/12)
Able to talk about it with someone else	73.3% (11/15)	41.7% (5/12)
Violence totally ceased	73.3% (11/15)	75% (9/12)
Violence diminished	20% (3/15)	16.7% (2/12)
Change in form of violence	46.7 (7/15)	45.5% (5/11)

## Discussion

### • Prevalence

The originality of this study lies in our decision to include consecutive patients consulting for an emergency visit, so that we had women and men, including Swiss and foreign patients, who took part in the study. We found rates of violence similar to those reported in other studies [6-8]. Our results show that men experience more community violence, and women more domestic violence, which is not very surprising. Doctors should have not only domestic but also community violence in mind when consulting with a patient.

We did not find a statistically significant difference between the Swiss and foreign patients' responses related to rates of violence. Not many studies have compared rates of violence among women and men, or among natives and foreigners. A United States national survey on intimate-partner violence [18] revealed that women experience more intimate-partner violence than men. The rates of intimate-partner violence vary significantly among women and men of different racial and ethnic backgrounds, but these differences diminish when other sociodemographic and relationship variables are controlled. A National Crime Victimization Survey in the United States found that women were more likely to be violently victimized by a friend/acquaintance (36.9%) or an intimate/other relative (28.8%), and men by a stranger (55.2%) [19]. A Swiss survey on the health of the population showed that 9% of the persons who were asked (8% of women and 10% of men) said that they had been a victim of at least one form of violence (verbal violence, physical violence, or offence against property) in the last 12 months [5]. More than half of them suffered verbal violence (5%), 1.5% suffered physical violence, and 3.7% reported an offence against property. In the case of physical violence, and more specifically sexual violence, women are more at risk of being victims than are men. We did not find reports on the proportion of foreign victims of violence in Switzerland, but police statistics document this number for criminal offences. Intentional homicides and bodily injuries are caused by foreigners in 53.3% of cases; they are also responsible for 2 out of 3 rape cases solved and 36% of other offenses against sexual integrity [3].

The detection of the domestic violence/community violence by physicians is low in our study (8%) as documented in the literature. One study found screening rates of only 13% for victims of acute intimate partner violence presenting to an emergency department [20]. Limited time, lack of education, ineffective intervention and the fact that healthcare workers often feel that they have nothing to offer are some of the common barriers to screen for domestic violence[21].

### • Follow-up

At follow-up, we noted that domestic violence had diminished or ceased for the majority of the patients we contacted. Victims and perpetrators did not differ in their responses, except that perpetrators tended to have more difficulty in talking about their violent behavior to people than the victims had in talking about what happened to them. It seems that the interview by the psychologist made it possible for the patients to initiate changes in their family life, resulting in decreased violence.

### • Limits

Patients were free to not participate, so we probably missed some who had a problem of violence. For example, one woman said she did not want to participate in the study because the subject concerned her and she was afraid that it would be too disturbing to talk about it. Also, as she was seeing a psychologist for this problem, she did not want to talk about it again.

We did not have a group control. This is a methodological limitation, but also an ethical necessity when working with people affected by violence. Victims of violence cannot be left without anyone to intervene.

## Conclusion

The rate of detection of domestic violence by physicians is insufficient, as is generally documented in the literature. The low physician suspicion/detection rate can be related to the lack of awareness that their patients are exposed to violence and their impression that it is not a relevant issue for that particular unit. Another reason could be the barriers to disclosure by the victim's feelings of shame, loyalty to the partner or intimidation by the perpetrator and fear of being not believed [22].

The originality of this study is that this is one of the few papers to assess prevalence of violence experiences in a primary care population clinic in Switzerland, including both Swiss nationals and foreign patients, and the assessment of the provider's understanding and recognition of his or her patient's exposure to violence.

The prevalence of violence is high, and domestic violence is more frequent than community violence. There was no statistically significant difference between the Swiss and foreign patients' responses related to rates of violence.

Finally, we noted that domestic violence had diminished or ceased at follow-up for the majority of the patients we contacted. Study participation was considered to be helpful by victims because they felt that the provider recognized their real problem and that they were not alone in this situation; the victim could also feel that the violence perpetrated against him or her was not deserved [23].

## Competing interests

The author(s) declare that they have no competing interests.

## Authors' contributions

Claire Morier-Genoud: conceived and designed the study, prepared the training and gave the lecture, collected the data by interviewing the patients, participated in the statistical analysis and the interpretation of the data, and wrote the manuscript.

Patrick Bodenmann: participated in the design and coordination of the study in the collection and interpretation of the data, and helped to draft the manuscript.

Bernard Favrat participated in the design of the study, did the statistical analysis, and participated in the interpretation of the data.

Marco Vannotti participated in the design of the study, gave the lecture, and participated in the interpretation of data.

All the authors read and approved the final manuscript.

## Pre-publication history

The pre-publication history for this paper can be accessed here:


